# Supplementary material for: Three-dimensional structure determination of protein complexes using matrix-landing mass spectrometry
Source: Nat Commun. 2022 Apr 27;13:2276. doi: 10.1038/s41467-022-29964-4 (PMC9046196; doi:10.1038/s41467-022-29964-4)
Supplement: Supplementary file 1 — Supplementary Information [file 41467_2022_29964_MOESM1_ESM.pdf]

Supplementary Information: 3D Structure Determination of Protein Complexes using Matrix-Landing Mass Spectrometry

Michael S. Westphall, Kenneth W. Lee, Austin Z. Salome, Jean M. Lodge, Timothy Grant, and Joshua J. Coon

SUPPLEMENTARY FIGURES

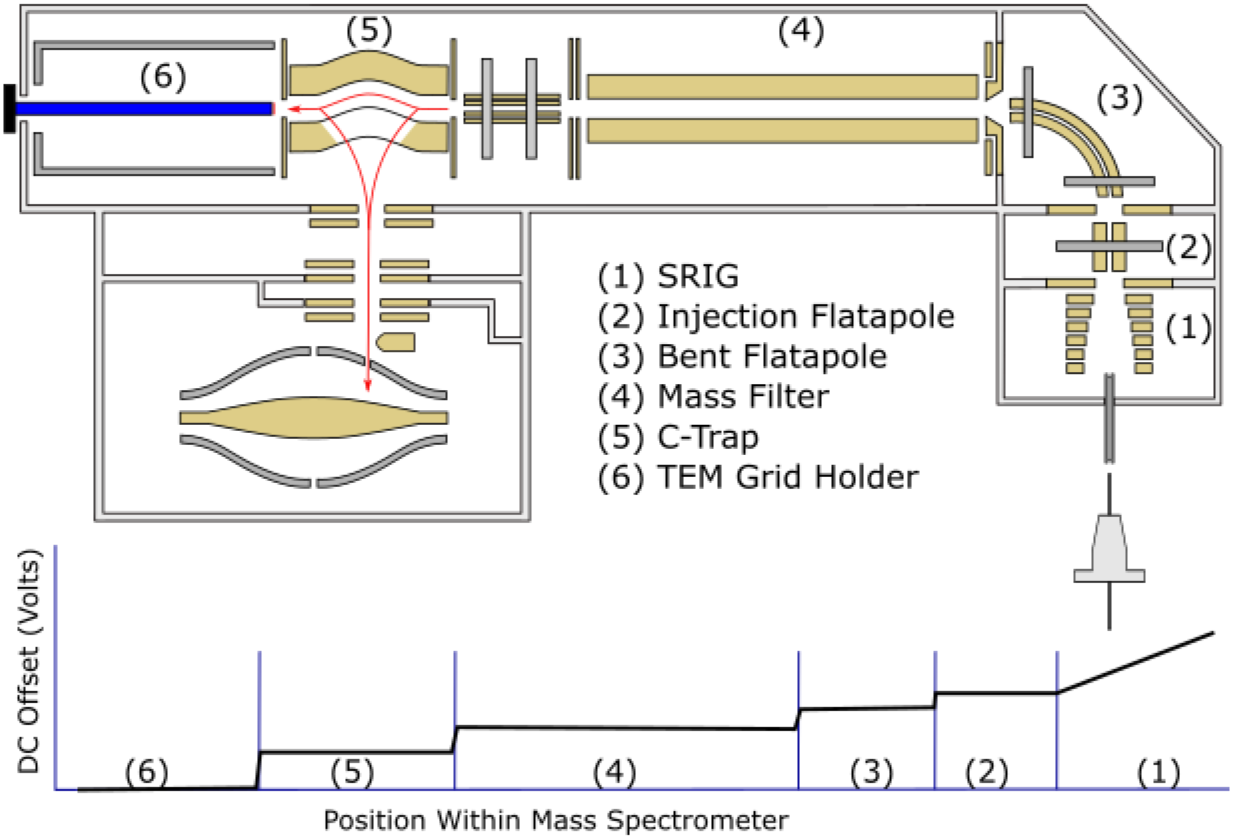

Supplementary Fig. 1 Orbitrap mass spectrometer modified to conduct matrix-landing of biomolecular ions. Ions are generated by nano-electrospray and initially injected into the stacked ring ion guide (SRIG, 1). The ions are then moved through the MS system and either injected into the Orbitrap mass analyzer for mass measurement or directed to the TEM grid (6). The DC offset potentials used during landing experiments is shown on the bottom panel.

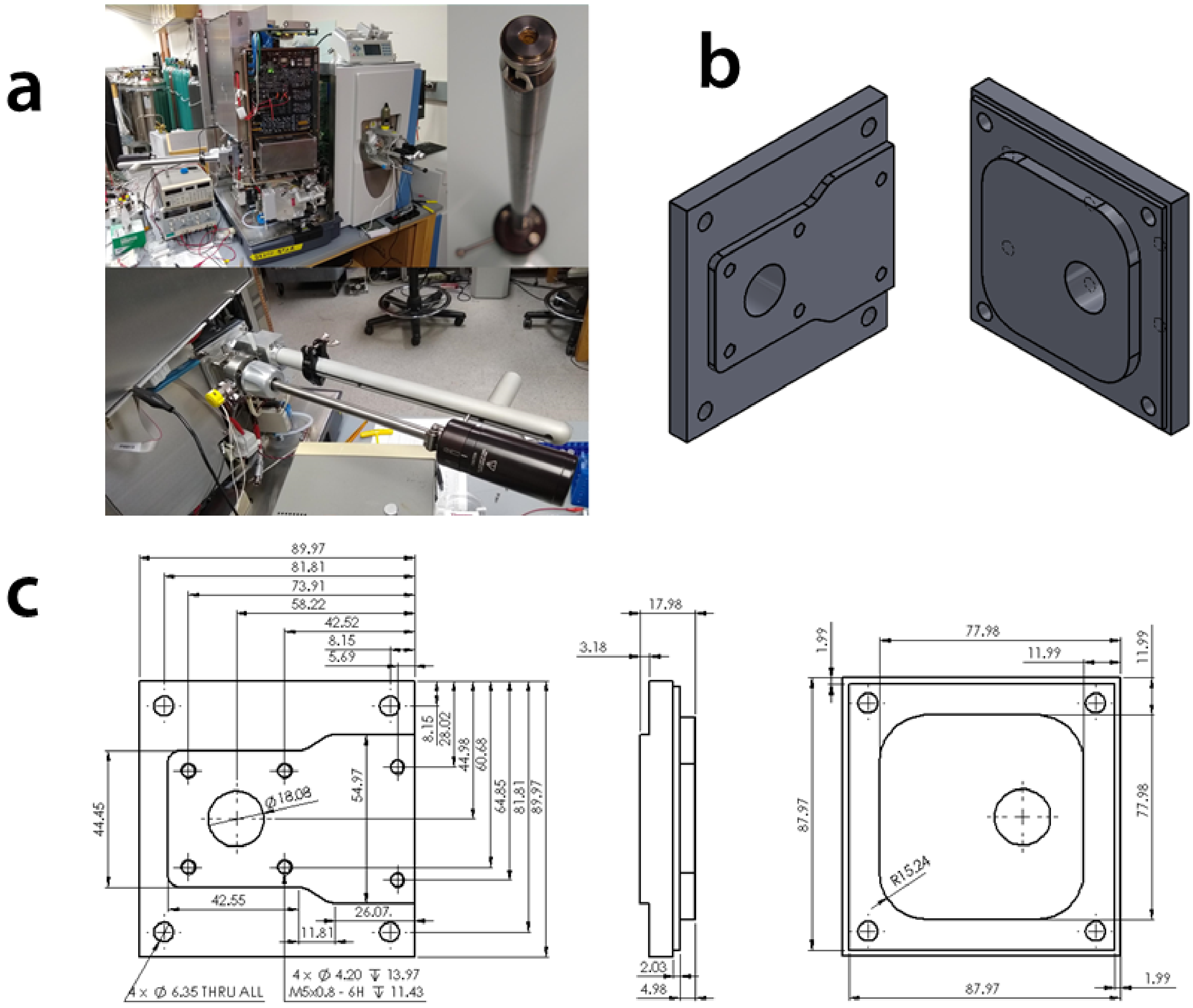

Supplementary Fig. 2 Thermo Velos Ion Trap ETD ion volume exchange components converted for TEM grid insertion into UHMR mass spectrometer. **(a)** Photo montage of the ETD components incorporated onto the UHMR. The ion volume insertion and removal tool are used to hold the TEM grid. **(b)** Incorporation of the inlet valve and guide bar assembly required a new endplate for the vacuum chamber of UHMR to be fabricated. The atmospheric side (left) and vacuum side (right) of the cover are displayed. **(c)** mechanical drawings required to duplicate the endplate. Dimensions are displayed in units of millimeters.

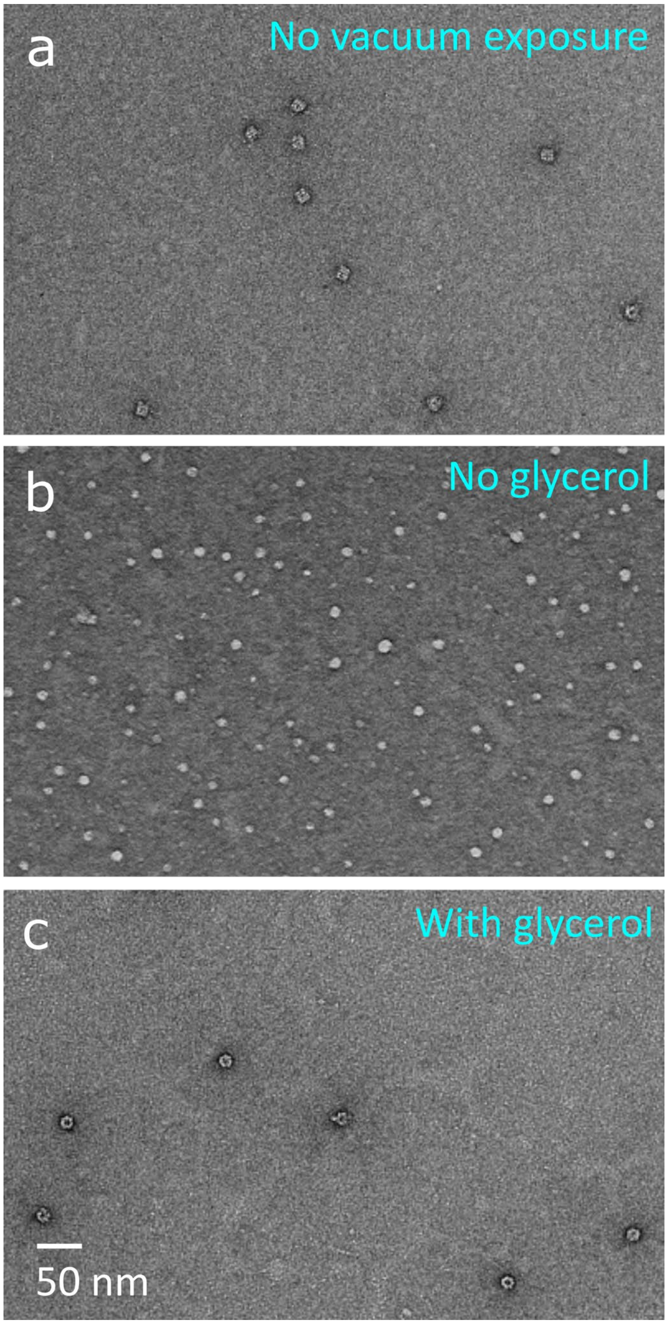

Supplementary Fig. 3 Negative stain TEM images of GroEL with and without vacuum exposure. (a) GroEL particles that were pipetted onto a bare TEM grid and immediately stained and imaged. **(b)** The same particles that were placed under vacuum (comparable to the landing apparatus pressures) for a period of 10 minutes prior to staining. GroEL particles are damaged following just 10 minutes of vacuum exposure. These images produce results strikingly similar to those of landed GroEL particles on bare TEM grids. **(c)** The same experiment except for (1) the TEM grid was treated with a thin film of glycerol and (2) the sample was exposed to vacuum for 60 minutes. Even after this extended vacuum exposure, intact GroEL particles were clearly visible. The images shown here were acquired from no fewer than five samples each.

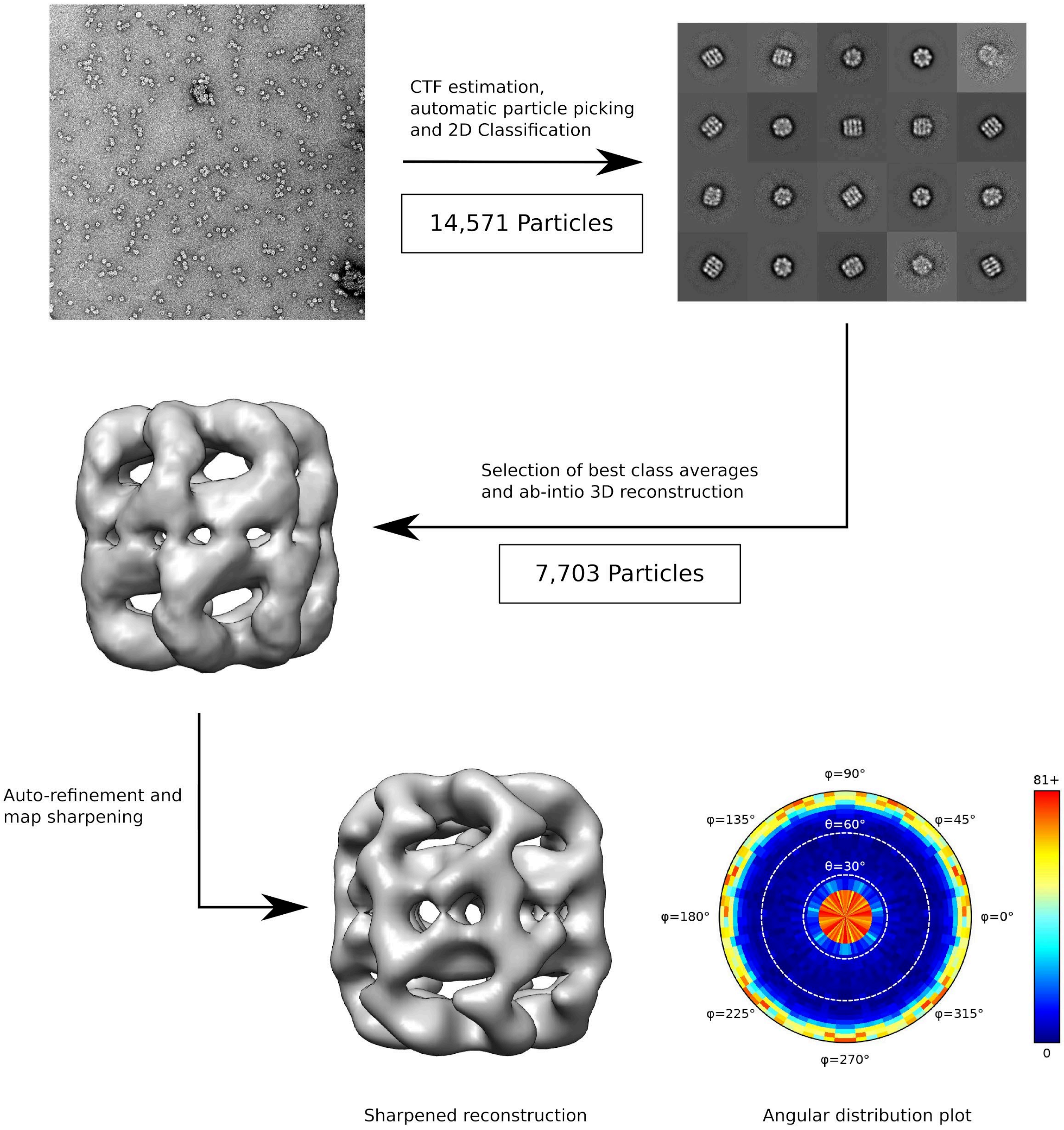

Supplementary Fig. 4 Electron microscopy data processing workflow. Flow chart of negative stain image processing for landed Gro-EL. A representative raw stain image and 2D class averages are shown. Automatic particle picking resulted in ~15,000 picked particles. After 2D classification and selection of the best classes ~7,000 particles were kept. Ab-inito 3D reconstruction followed by auto-refinement and map sharpening resulted in the final 3D reconstruction which is shown along with a plot demonstrating the angular distribution of the particles.

SUPPLEMENTARY TABLES

Supplementary Table 1 Cryo-EM data collection conditions

	3D Structure Determination of GroEL Protein Complexes using Matrix-Landing Mass Spectrometry (EMDB ID: EMD-26222)
**Data collection and processing**	
Magnification	30,000
Voltage (kV)	120
Electron exposure (e–/Å^2)	Negative stain, not measured
Defocus range (μm)	1 to 3
Pixel size (Å)	3.4
Symmetry imposed	D7
Initial particle images (no.)	14,571
Final particle images (no.)	7,703
Map resolution (Å)	14
FSC threshold	0.143
